# Optimization of Ultrasound-Assisted Extraction of Crude Oil from Winter Melon (*Benincasa hispida*) Seed Using Response Surface Methodology and Evaluation of Its Antioxidant Activity, Total Phenolic Content and Fatty Acid Composition

**DOI:** 10.3390/molecules171011748

**Published:** 2012-10-08

**Authors:** Mandana Bimakr, Russly Abdul Rahman, Farah Saleena Taip, Noranizan Mohd Adzahan, Md. Zaidul Islam Sarker, Ali Ganjloo

**Affiliations:** 1 Department of Food Technology, Faculty of Food Science and Technology, Universiti Putra Malaysia, 43400 Serdang, Selangor, Malaysia; E-Mails: mandanabimakr@yahoo.com (M.B.); noraadzahan@food.upm.edu.my (N.M.A.); aganjloo@yahoo.com (A.G.); 2 Department of Process and Food Engineering, Faculty of Engineering, Universiti Putra Malaysia, 43400 Serdang, Selangor, Malaysia; E-Mail: saleena@eng.upm.edu.my; 3 Halal Product Research Institute, Universiti Putra Malaysia, 43400 Serdang, Selangor, Malaysia; 4 Department of Pharmaceutical Technology, Faculty of Pharmacy, International Islamic Universiti Malaysia, 25200 Kuantan, Pahang, Malaysia; E-Mail: zaidul@iium.edu.my; 5 Department of Food Science and Technology, Faculty of Agriculture, Zanjan University, Zanjan P.O. Box 313, Iran

**Keywords:** ultrasound-assisted extraction, winter melon, antioxidant activity, total phenolic content, fatty acid composition

## Abstract

In the present study, ultrasound-assisted extraction of crude oil from winter melon seeds was investigated through response surface methodology (RSM). Process variables were power level (25–75%), temperature (45–55 °C) and sonication time (20–40 min). It was found that all process variables have significant (*p* < 0.05) effects on the response variable. A central composite design (CCD) was used to determine the optimum process conditions. Optimal conditions were identified as 65% power level, 52 °C temperature and 36 min sonication time for maximum crude yield (108.62 mg-extract/g-dried matter). The antioxidant activity, total phenolic content and fatty acid composition of extract obtained under optimized conditions were determined and compared with those of oil obtained by the Soxhlet method. It was found that crude extract yield (CEY) of ultrasound-assisted extraction was lower than that of the Soxhlet method, whereas antioxidant activity and total phenolic content of the extract obtained by ultrasound-assisted extraction were clearly higher than those of the Soxhlet extract. Furthermore, both extracts were rich in unsaturated fatty acids. The major fatty acids of the both extracts were linoleic acid and oleic acid.

## 1. Introduction

*Benicasa hispida* L. (*Cucurbitaceae*), is variously named winter melon, white gourd, ash pumpkin, tallow gourd, white pumpkin, ash gourd, wax gourd, gourd melon and Chinese watermelon or Chinese preserving melon in English [1]. The *Cucurbitaceae* family is mostly distributed around the tropical regions and the winter melon, which has been cultivated for at least 2,000 years, originated from south-east Asia [2]. This fruit is large and seedy with white colored and spongy flesh. Depending on the shape, type and maturity of the fruit, the seeds, which are smooth and white to yellowish-colored, fill the centre of the fruit [2,3]. Index of Nutritional Quality (INQ) data shows that *Benicasa hispida* is valued as a high quality vegetable [2]. Several investigations on the biologically active components of *Benincasa* species have proven its antioxidant activity on different tissues like liver and brain [4]. To the best of our knowledge, studies on chemical composition of seed oil from *Benincasa hispida* and its antioxidant activity have not been reported yet.

Extraction is a major step for the isolation, identification and use of valuable compounds from different plants [5]. The Soxhlet method which was developed by von Soxhlet in 1879 has been performed as a standard method for extraction of valuable compounds from different plant sources. This technique is not always acceptable for an extraction due to the fact that this process is very slow and degradation of targeted compounds is common [6]. Numerous studies have been carried out to develop novel extraction processes which are applicable to various compounds [6,7]. In recent years, ultrasound-assisted extraction has received considerable attention for the recovery of different compounds from different sources [8,9,10]. This technique is attractive because of its simplicity and low equipment cost compared with other extraction techniques such as supercritical fluid or microwave-assisted extraction [11]. Moreover, other advantages include drastically reduced processing time, consumption of less energy and reduced thermal degradation effects [12]. The higher efficiency of ultrasound-assisted extraction is attributed to disruption of cell walls, particle size reduction and enhanced mass transfer of the cell content via cavitation bubble collapses [13,14]. Various applications of ultrasound in extraction of bioactive compounds from plant materials were reviewed by Vinatoru [15]. So far, there is no report on ultrasound-assisted extraction of crude oil from *Benincasa hispida* seed. Furthermore, the application of ultrasound in food technology is reviewed by Chemat *et al*. [16].

Optimization of the experimental conditions is a critical step in developing a successful ultrasound-assisted extraction process due to the effect of various process variables such as ultrasound power, process temperature and sonication time on the extraction efficiency [11]. Response surface methodology (RSM) is an effective statistical method for optimizing experimental conditions and investigation of critical processes, while at the same time reducing the number of experimental trials [17]. Therefore, the objectives of this study were to investigate the effect of process variables including power level, temperature and sonication time on crude extract yield (CEY). RSM was employed to optimize extraction conditions in order to obtain the maximum crude yield. Furthermore, the antioxidant activity, total phenolic content and fatty acid composition of the extract obtained under optimized conditions were determined and then compared with those obtained by the Soxhlet method. 

## 2. Results and Discussion

### 2.1. Effect of the Process Variables on Crude Extract Yield 

**Figure 1:**
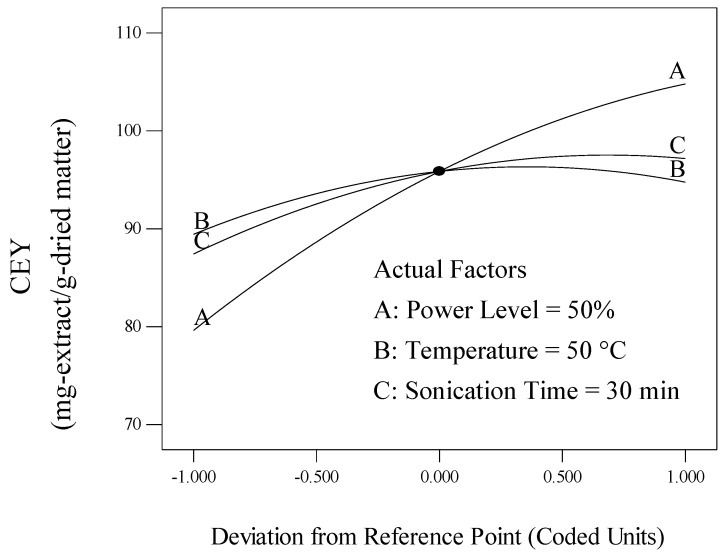
Perturbation graph showing the effect of process variables on crude extract yield.

The individual effect of process variables, including power level (A), temperature (B) and sonication time (C), on the CEY was found by perturbation plot. A perturbation plot does not show the effect of interactions and it is like one factor-at-a-time experimentation. The perturbation plot helps to compare the effect of all process variables at a particular point in the design space. The response is plotted by changing only one factor over its range while holding of the other factors constant. A steep slope or curvature in a factor shows that the response is sensitive to that factor. A relatively flat line shows insensitivity to change in that particular factor. Therefore, it was revealed that CEY is more sensitive to power level than sonication time and temperature. The perturbation plot for the CEY is shown in Figure 1. 

### 2.2. Optimization of Ultrasound-Assisted Extraction Conditions and Verification of the Model 

The experimental design and related experimental results are presented in Table 1. The crude yield ranged from 65.33 to 108.20 mg-extract/g-dried matter. A second-order polynomial model was rendered by multiple linear regression analysis of the experimental data. The regression coefficients and significant probabilities of linear, quadratic and interaction effects of process variables are shown in Table 2. Probability values (*p*-value) revealed that all linear, quadratic and interaction terms, except interaction of power level-temperature (X_1_X_2_), had significant (*p* < 0.05) effects on the CEY. Therefore, the final reduced model was obtained by elimination of the non-significant term from the initial model as follows:

CEY = 95.86 + 12.59 **X_1_** + 2.66 **X_2_** + 4.87 **X_3_** + 2.11 **X_1_X_3_** + 0.76 **X_2_X_3_** − 3.65 **X_1_^2^** − 3.76 **X_2_^2^** − 3.56 **X_3_^2^** (1)

where X_1_, X_2_ and X_3_ represent power level, temperature and sonication time, respectively. According to Table 2, the *p*-value of the final reduced model was less than 0.05 which revealed that model fitness was significant. Moreover, the lack of fit test was non-significant (*p* > 0.05), indicating that the model could adequately fit the experiment data (Table 2).

**Figure 2:**
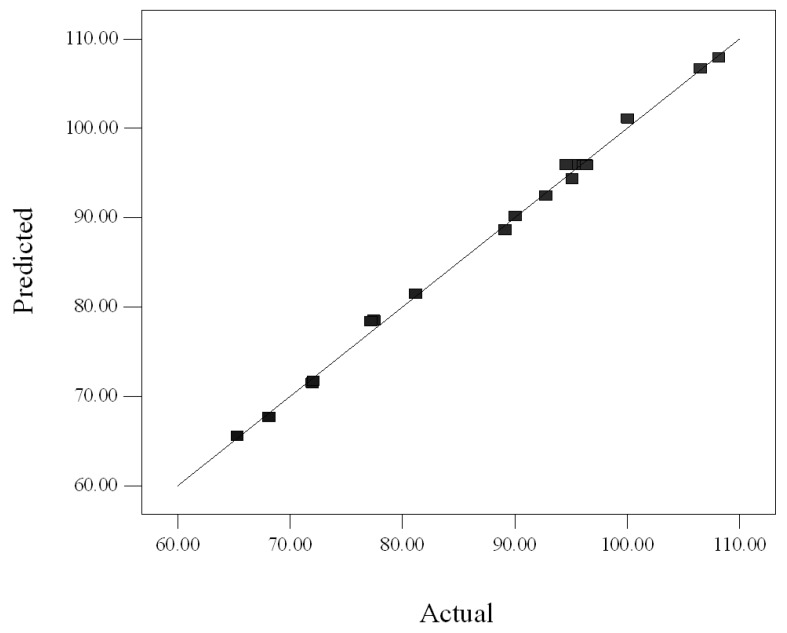
Plot of predicted crude extract yield related with experimental values.

Figure 2 shows that the predicted values were very close to the actual values. Indeed, the high value of R^2^ (0.997) confirmed the true behavior of the system which defined by the regression model. In addition, the suitability of the rendered model was supported by the closeness of adjusted-R^2^ (0.995) to 1 presenting a high degree of correlation between the experimental and predicted values. The E value was also calculated and is presented in Table 2. The small E value (0.55%) suggested that the obtained model was acceptable.

**Figure 3:**
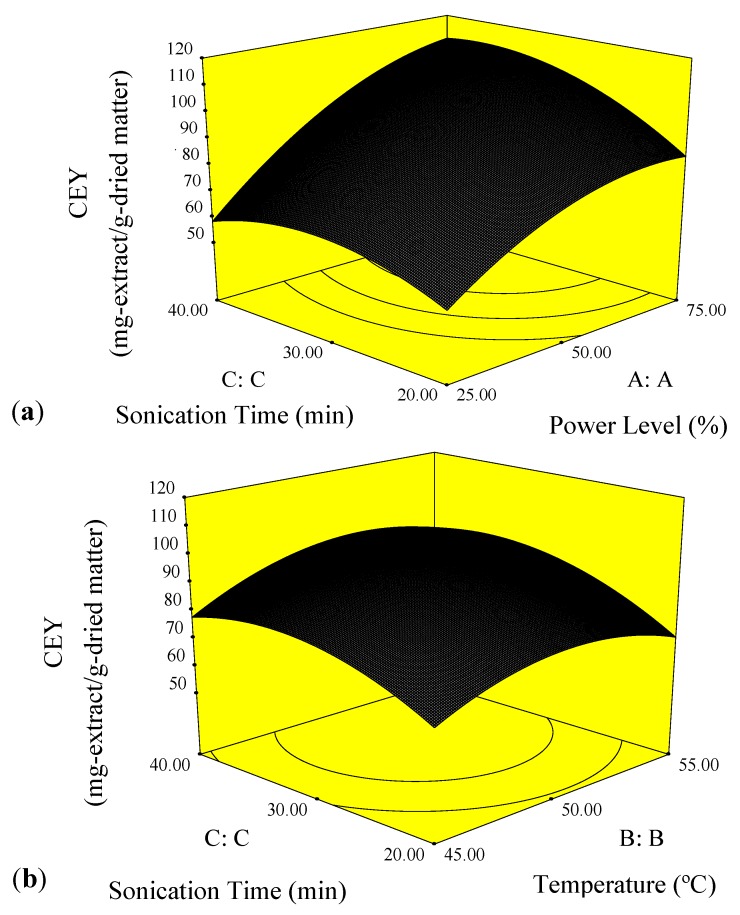
Response surface plots for CEY (mg-extract/g-dried matter) as a function of: (**a**) power level (%) and sonication time (min) (**b**) temperature (°C) and sonication time (min).

Figure 3 presents the three dimensional response surface plots which are highly recommended for the graphical interpretation of the interaction effect of process variables on the response [18]. Figure 3a illustrates the effect of power level and sonication time on the CEY at a constant temperature of 50 °C. At a fixed power level, the CEY increased when sonication time was extended to 35 min whereas it was diminished by 40 min of sonication time. It was reported that 40 min of sonication time was sufficient to complete the extraction process of epimedin C from fresh leaves of *Epimedium* [19] and anthocyanins from mulberry [20]. It is worthy of note that the ultrasound-assisted extraction mechanism consists of two main stages. First, “washing” which is dissolution of soluble compounds on surfaces of the plant matrix. Secondly, “slow extraction” which defined as mass transfer of the solute from the plant matrix into the solvent by diffusion and osmotic processes [21]. These two phenomena were clearly observed in Figure 3a,b. Washing is happening at the beginning of the extraction with a rapid increase. After that, the slow extraction is observed by a low raise in crude yield. Moreover, it was found out that increase of power level (X_1_) resulted in increase of CEY. Higher amplitude of ultrasound could have damaged more cell walls releasing more crude oil to the solvent [22]. This finding is in agreement with those obtained by Hossain *et al*. and Zou *et al*. [23,24].

Figure 3b presents the effect of temperature and sonication time on the CEY at a constant power level of 50%. At a fixed sonication time, the crude yield increased with the increase of temperature (X_2_) followed by a decline with the further increase of the extraction temperature. Our finding was in agreement with those obtained by Zhang *et al*. [19] and Zhang *et al*. [25]. This reduction could be due to adverse effect of temperature on process improvement as a result of decrease in number of acoustic cavitation bubbles generated by ultrasonic waves [25]. 

A numerical optimization was performed through desirability function method to determine the optimum level of process variables leading to maximum CEY. The optimal conditions were determined as 65% power level, 52 °C temperature and 36 min sonication time. The rechecking experiment was performed in triplicate according to the optimal conditions in order to compare the predicted result with the practical value. The mean value of 108.36 mg-extract/g-dried matter obtained from real experiment which was very close to the predicted result (108.62 mg-extract/g-dried matter) indicating the validity and adequacy of response model to reflect the expected optimization.

### 2.3. Comparison of Ultrasound-Assisted Extraction with Soxhlet Method

**Figure 4:**
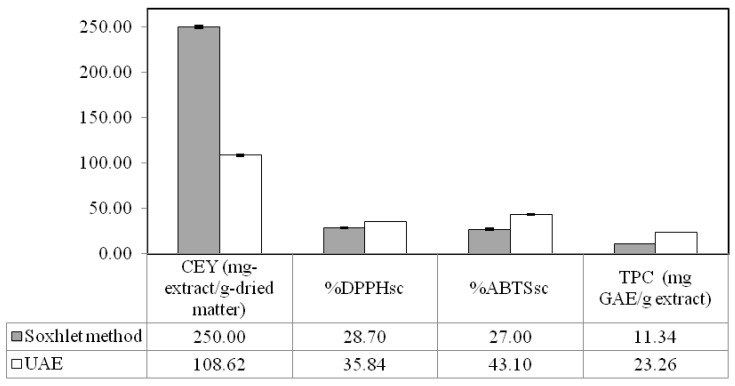
Comparison the quality of extracts obtained by ultrasound-assisted extraction and Soxhlet method.

**Figure 5:**
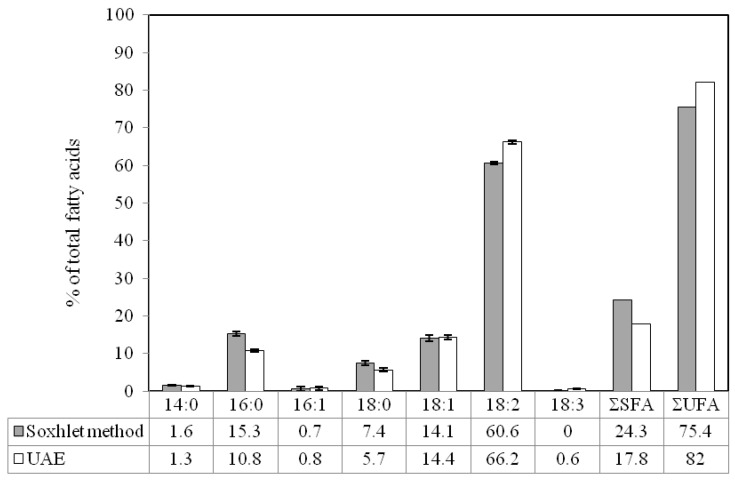
Fatty acid composition of extracts obtained using optimized ultrasound-assisted extraction and Soxhlet method.

The Soxhlet method, as mentioned in the introduction, has been performed as a standard method for extraction of valuable compounds from different plant sources. In this study, optimized ultrasound-assisted extraction was compared with the Soxhlet method in terms of CEY, antioxidant activity, total phenolic content and fatty acid composition of the extracts. The results are presented in Figure 4 and Figure 5. It was found that the CEY obtained using optimized ultrasound-assisted extraction method was definitely lower than that obtained by the Soxhlet method. It should be kept in mind that the conventional extraction method required 6 h for release of around 250 mg-extract/g-dried matter crude yield at an operating temperature of 78 °C and a solvent to solute proportion of 30:1 whereas the ultrasound-assisted extraction method required only 36 min for release of 108 mg-extract/g-dried matter crude yield at lower temperature and proportion of solvent to solute. This result is in agreement with that reported by Jadhav *et al*. [27] for extraction of vanillin from vanilla pods. 

Obviously, the extract obtained by optimized ultrasound-assisted extraction method have higher quality in terms of DPPH**˙**, ABTS**˙^+^** and total phenolic content than that obtained by conventional method (Figure 4). This could be due to the thermal degradation of bioactive compounds which contributed to the antioxidant activity of extract. It was expected that these compounds are protected from thermal degradation by using an extraction technique with lower temperature and shorter extraction time. Considering the thermal sensitivity of phenolic compounds, it is obvious that these compounds have been degraded by Soxhlet method because of the high temperature and long extraction time which is common in the Soxhlet method. 

The fatty acid composition of extracts obtained using optimized ultrasound-assisted extraction and Soxhlet method is presented in Figure 5. It was observed that both of the extracts were very rich in unsaturated fatty acids (up to 82% for ultrasound-assisted extraction and 75% for Soxhlet method). The major unsaturated fatty acids which detected in extracts obtained using the Soxhlet method and UAE were linoleic acid (60.6 and 66.2% of total fatty acids, respectively) and oleic acid (14.1 and 14.4% of total fatty acids, respectively). α-Linolenic acid, which is a kind of omega-3 fatty acid, was detected in extract obtained by the optimized ultrasound-assisted extraction method. Omega-3 fatty acids have been introduced as effective dietary supplements for the management of different diseases by enhancing the mechanisms of antioxidant defense [28]. On the other hand, a relatively low content of saturated fatty acids (SFA) was detected in both extracts (Figure 4), but it should be mentioned that higher temperature seems to be more favorable for extraction of SFA [29]. 

## 3. Experimental

### 3.1. Material 

Whole winter melons (*Benincasa hispida* L.) were purchased from a local market in Serdang, Selangor, Malaysia. Fruits were chosen at commercial maturity according to their similarity of color, size and absence of surface defects. The fruits were cut, and then seeds were separated manually and washed under tap water. Seeds were dried at 40 °C in a ventilated oven (1350FX, Cornelius, OR, USA) for 24 h and then stored at an ambient temperature in the dark. The seeds were ground in a grinder mill (MX-335, Panasonic, Shah Alam, Malaysia) for 10 s to produce a powder with an approximate size of 1.5–2.5 mm. 

### 3.2. Chemicals and Reagents

Carbon dioxide (CO_2_, SFE grade) contained in a dip tube cylinder was purchased from MOX-Linde Gases Sdn. Bhd. (Petaling Jaya, Malaysia). Analytical grade ethanol and *n*-hexane were obtained from Scharlau (Port Adelaide, Australia). Sodium methoxide, potassium persulphate, catechin, 2,2′-azinobis(3-ethylbenzothiazoline-6-sulphonic acid) diammonium salt (ABTS**˙^+^**), 1,1-diphenyl-2-picrylhydrazyl (DPPH**˙**), gallic acid and Folin-Ciocalteu reagent (FCR) were purchased from Fisher (Pittsburgh, PA, USA). Fatty acid methyl ester (FAME) standards were obtained from Sigma-Aldrich (St. Louis, MO, USA). All chemicals were either of chromatography or analytical grade.

### 3.3. Ultrasound-Assisted Extraction 

**Figure 6:**
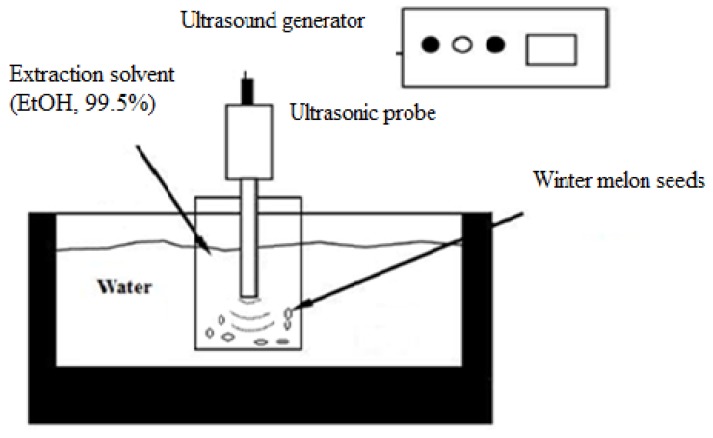
Scheme of experimental set-up for ultrasound-assisted extraction [30].

In present study, a 500 W ultrasound equipment (Sonics and Materials Inc., Model VC505, Danbury, CT, USA) with a titanium ultrasonic probe (13 mm diameter) was used for ultrasound-assisted extraction of crude oil from *Benincasa hispida* seeds. The nominal frequency was 20 kHz. Ground seed (about 5 g) was placed in a 100 mL beaker containing ethanol as a food grade solvent which is recommended by the US Food and Drug Administration for extraction purposes. The solid/solvent ratio was 1:10 (g/mL). The beaker placed in a temperature controlled water bath (Memmert WNE14. Memmert GmbH Co. KG, Schwabach, Germany). Extraction was carried out at temperatures ranging from 45 to 55 °C. Temperature was verified with a digital thermometer (Ellab CTD-85, Ellab, Hilleroed, Denmark) and a thermocouple (1.2 mm needle diameter constantan type T) and no significant increase in temperature (below 2 °C) was detected due to circulation of water in water bath during extraction. The applied power levels were adjusted to 25, 50 and 75% of the maximal equipment power (500 W), corresponding to 125, 250 and 375 W through the variation of amplitude of piezocrystals. The corresponding ultrasound intensities were 94, 189 and 284 W/cm^2^. The immersed samples in extraction solvent were subjected to ultrasonic waves for 20 to 40 min. The input range of the selected variables was determined by preliminary experiments. After extraction, the extracts were filtered through the Whatman No. 1. filter paper. Then, ethanol was removed from the extracts by evaporation under vacuum at 40 °C using a rotary evaporator (Eyela, A-1000S, Koishikawa Bunkyo-ku, Japan). Subsequently, the residual solvent was removed by drying in an oven at 40 °C for 1 hr and flushing with 99.9% nitrogen. The scheme of experimental set-up was presented in Figure 6. 

### 3.4. Soxhlet Method

Ground *Benincasa hispida* seed (about 5 g) was put into extraction thimble and covered with wool. Then the thimble was transferred into a Soxhlet apparatus. Extraction was performed with ethanol (99.5%, 150 mL) for 6 h. The temperature of extraction corresponded with the boiling point of the solvent in use. After extraction, solvent was removed under vacuum at 40 °C using a rotary evaporator (Eyela). Subsequently, the residual solvent was removed by drying in an oven at 40 °C for 1 h and flushing with 99.9% nitrogen. 

### 3.5. Crude Extract Yield Measurement

The extracts were weighed gravimetrically using a Mettler Toledo analytical balance (±0.0001 g) (Mettler Toledo GmbH, Greinfensee, Switzerland) and then the CEY was calculated according to the following equation:

$$CEY=\frac{m_{e}}{m_{s}}\times 1000$$

where m_e_ is the crude extract mass (g) and m_s_ is the extracted sample mass (g). The measurement was performed in triplicate and the mean values of CEY were expressed as mg-extract/g-dried matter.

### 3.6. Determination of Radical Scavenging Activity 

The extracts *of Benincasa hispida* seeds were subjected to antioxidant activity analysis using DPPH**˙** and ABTS**˙**^+^ free radical scavenging assays. All determinations were done in triplicate and expressed as means ± Standard Deviation.

#### 3.6.1. Determination of DPPH**˙** Radical Scavenging Activity

This assay was carried out as described by Zengin *et al.* [31] with some modifications. A total of 0.1 mg/mL of the extracts and synthetic antioxidant (catechin) in the ethanol were added into an ethanolic solution of DPPH**˙** (3 mL, 6 × 10^−5^ M). The mixture was vortexed for 20 s at room temperature. Absorbance measurements at 515 nm commenced immediately in a 1 cm quartz cell after 1 min up to 60 min with 10 min intervals using a UV-260 visible recording spectrophotometer (Thermo 4001/4 UV–Vis Spectrophotometer, Thermo Fisher Scientific, West Palm Beach, FL, USA). The blank test was conducted with 0.1 mL ethanol instead of extracts and the absorbance was recorded as A_blank_. The inhibition percent of DPPH**˙** which was scavenged (%DPPH_sc_) was calculated according to the following equation:

% DPPH_sc_ = 100 × (A_blank_ − A_sample_)/A_blank_

where A_blank_ and A_sample_ are the absorbance values of the blank and of the tested samples, respectively, checked after 60 min. 

#### 3.6.2. Determination of ABTS**˙^+^** Radical Scavenging Activity 

The 2,2´-azinobis(3-ethylbenzothiazoline-6-sulphonic acid) diammonium salt (ABTS**˙^+^**) assay was carried out according to the method of Cai *et al.* [32]. The ABTS**˙^+^** radical solution was prepared by mixing 7 mM ABTS and 2.45 mM potassium persulphate, and incubating the mixture in the dark at room temperature for 16 h. The ABTS**˙^+^** solution was then diluted with 80% (v/v) ethanol to obtain an absorbance of 0.70 at 734 nm. ABTS**˙^+^** solution (3.9 mL) was added to sample (0.1 mg/mL) and mixed vigorously. The absorbance of the mixtures at room temperature was recorded immediately using UV-260 visible recording spectrophotometer (Thermo 4001/4 UV–Vis Spectrophotometer) at 734 nm for 10 min at 2 min intervals. The blank test was conducted with ethanol instead of extracts and the absorbance was recorded as A_blank_. The inhibition percent of ABTS**˙^+^** which was scavenged (%ABTS_sc_) was calculated using the following equation:

%ABTS_sc_ = (A_blank_ – A_sample_) × 100/A_blank_

where A_blank_ and A_sample_ are the absorbance values of the blank and of the tested samples, respectively.

### 3.7. Determination of Total Phenolic Content 

The total phenolic content (TPC) of the *Benincasa hispida* seed extracts was determined using Folin-Ciocalteu reagent (FCR) according to the procedure reported by Singleton *et al.* [33] with some modifications. This method is based on measuring color change caused by reduction of the Folin-Ciocalteu reagent by phenolates in the presence of sodium carbonate. Extract (about 10 mg) was dissolved in deionised water (1 mL). This solution was mixed with Folin-Ciocalteu reagent (diluted 10 fold with distilled water, 1 mL). The solution was kept at room temperature for 5 min and then 60 mg/mL of aqueous carbonate sodium (Na_2_CO_3_) solution (7.5 mL) was added. The color change was determined by scanning the wavelength at 765 nm (Thermo 4001/4 UV–Vis Spectrophotometer) since maximum absorbance was obtained. TPC of the extract was determined as mg gallic acid equivalent using the standard curve prepared at different concentrations of gallic acid (25–500 ppm) and reported as mg GAE/g extract. The determination was carried out in triplicate and expressed as means ± Standard Deviation. 

### 3.8. Preparation of Fatty Acid Methyl Esters 

Samples were brought to a temperature of 50–60 °C and homogenized thoroughly before taking a test sample in order to obtain the fatty acid methyl esters (FAMEs). An aliquot of the test sample (100 µL) was mixed with *n*-hexane (1 mL) in a 2 mL vial. An aliquot of sodium methoxide (1 µL, 1% w/v) was added to the vial which was mixed vigorously using a vortex mixer. The mixture first became clear and then turbid as sodium glyceroxide was precipitated. After a few minutes, the clear upper layer of methyl ester was pipetted off and injected into the gas chromatograph (GC) for further analysis. 

### 3.9. Gas Chromatography Analysis 

Fatty acids composition analysis was performed in a Hewlett-Packard 6890 gas chromatography (Wilmington, DE, USA), equipped with a flame ionization detector (FID) and a BPX70 (30 m × 0.25 mm × 0.25 µm, Victoria, Australia) GC column. Oven temperature was programmed isothermally to 115 °C during 2 min, then was raised at 4 °C/min to 163 °C and then at 1 °C/min to 170 °C. Finally, temperature increased to 200 °C at 10 °C/min and held at this temperature for 2 min. Helium was used as a carrier gas which flowed at a rate of 1 mL/min. The injection volume was 1 µL. Standard methyl esters of fatty acids were used as authentic samples. The fatty acids determination was accomplished by comparing with standards and was valued by the area percentage of each fatty acid. The fatty acid determination was performed in triplicate for each sample and expressed as means ± Standard Deviation. 

### 3.10. Experimental Design and Statistical Analysis 

Response surface methodology (RSM) was applied to optimize the process variables including power level (25–75%), temperature (45–55 °C) and sonication time (20–40 min) to achieve the highest amount of crude oil from *Benincasa hispida* seeds. A central composite design (CCD) with axial points was used for designing the experimental data. This generated 20 treatments with six replications at the centre points to estimate the repeatability of the method (Table 1). The effect of unexplained variability induced by extraneous factors on the observed responses was minimized by randomizing the order of experiments. Blocks are assumed to have no impact on the nature and shape of the response surface. The following second-order polynomial model was fitted to the data:

Y_i_ = β_0_ + β_1_X_1_ + β_2_X_2_ + β_3_X_3_ + β_11_X_1_^2^ + β_22_X^2^_2_ + β_33_X_3_^2^ + β_12_X_1_X_2_ + β_13_X_1_X_3_ + β_23_X_2_X_3_

where Y_i_ is predicted response, β_0_ is offset term, β_1_, β_2_ and β_3_ are the regression coefficients for linear effect terms, β_11_, β_22_ and β_33_ are quadratic effects and β_12_, β_13_ and β_23_ are interaction effects. In this model, X_1_, X_2_ and X_3_ represent power level, temperature and sonication time, respectively. The significant terms (*p* < 0.05) in the model were found by analysis of variance (ANOVA) based on *p*-value. The terms statistically found non-significant (*p* > 0.05) were dropped from the initial model and the experimental data was refitted only to significant (*p* < 0.05) variables in order to obtain the final reduced model [34].

The three-dimensional response surface plot was generated for the graphical interpretation of the interaction effect of independent variables on the response. Numerical optimization was carried out to predict the exact optimum level of independent variables leading to the desirable response goal. The model adequacy was determined using model analysis, lack of fit test, coefficient of determination (R^2^) and adjusted-R^2^ [35]. Furthermore, experimental data were compared with predicted values (method validation) in order to verify the adequacy of final reduced model. In addition, the quality of fit between the experimental and predicted data was determined according to value of the mean relative deviation modulus (E). The criteria can be calculated as follows:

$$E\;(\%)=\frac{100}{n}\sum_{i=1}^{n}\frac{\left|Vexp-Vpre\right|}{Vexp}$$

where V_exp_ and V_pre_ are the experimental and predicted values, respectively, n is the number of experimental data. A model is considered acceptable if E value is less than 10% [36]. The experimental design matrix, data analysis, regression coefficients, generation of 3D graph and numerical optimization procedure were created using Design Expert Version 8.0.7 software (Stat-Ease Inc., Minneapolis, MA, USA, trial version). 

## 4. Conclusions 

In this study, response surface methodology with a central composite design (CCD) was applied to investigate the ultrasound-assisted extraction of crude oil from *Benincasa hispida* seed. The experimental results showed that all three process variables, including power level, temperature and sonication time, contributed to the extraction of crude oil. It was found that ultrasound power is the most significant variable among the process variables studied. An empirical quadratic polynomial correlation has been proposed to estimate the optimum operating condition of the process. The highest crude extract yield (108.62 mg-extract/g-dried matter) was obtained when the extraction process was carried out at 65% power level, 52 °C temperature and sonication time of 36 min. The results of the comparative study revealed that the CEY of ultrasound-assisted extraction was lower than that obtained by the Soxhlet method, whereas the extract obtained using the ultrasound-assisted technique had higher quality in terms of antioxidant activity and total phenolic content. Furthermore, it was revealed that the both extracts were rich in unsaturated fatty acids, with the majority corresponding to linoleic acid and oleic acid. Therefore, it may be said that plant sources like winter melon (*Benincasa hispida*) may provide new natural products to the food industry with safer and better antioxidants that provide good protection against oxidative damage which occurs both in the body and in the processed food. In addition, it could be suggested that ultrasound-assisted extraction is an effective and indeed feasible method for the extraction of crude oil rich in valuable compounds from the *Benincasa hispida* seed*.*

## Figures and Tables

**Table 1:** Experimental design (coded and uncoded levels) and results of response variable.

Run	Process Variables			CEY (mg-extract/g-dried matter)
	Power Level (%) (X_1_)	Temperature (°C) (X_2_)	Sonication Time (min) (X_3_)	
1	34.69 (−1)	46.94 (−1)	36.12 (+1)	72.10
2	50.00 (0)	50.00 (0)	30.00 (0)	95.44
3	50.00 (0)	50.00 (0)	30.00 (0)	96.24
4	65.31 (+1)	53.06 (+1)	23.88 (−1)	92.81
5	50.00 (0)	50.00 (0)	30.00 (0)	96.43
6	65.31 (+1)	46.94 (−1)	23.88 (−1)	89.18
7	34.69 (−1)	46.94 (−1)	23.88 (−1)	68.18
8	50.00 (0)	50.00 (0)	30.00 (0)	94.60
9	34.69 (−1)	53.06 (+1)	36.12 (+1)	77.50
10	34.69 (−1)	53.06 (+1)	23.88 (−1)	72.00
11	65.31 (+1)	46.94 (−1)	36.12 (+1)	100.1
12	65.31 (+1)	53.06 (+1)	36.12 (+1)	108.2
13	50.00 (0)	50.00 (0)	20.00 (−1.63)	77.23
14	75.00 (+1.63)	50.00 (0)	30.00 (0)	106.5
15	25.00 (−1.63)	50.00 (0)	30.00 (0)	65.33
16	50.00 (0)	55.00 (+1.63)	30.00 (0)	90.11
17	50.00 (0)	50.00 (0)	40.00 (+1.63)	95.15
18	50.00 (0)	50.00 (0)	30.00 (0)	96.43
19	50.00 (0)	45.00 (−1.63)	30.00 (0)	81.20
20	50.00 (0)	50.00 (0)	30.00 (0)	96.17

**Table 2:** Analysis of variance (ANOVA) and coefficients of the final reduced regression equation.

Source	df	CEY (mg-extract/g-dried matter)		
		Coefficient	Sum of Squares	*p*-value
Model	8		3,028.59	<0.0001
Const.		95.86		
X_1_	1	12.59	2,112.73	<0.0001
X_2_	1	2.66	94.52	<0.0001
X_3_	1	4.87	316.81	<0.0001
X_1_^2^	1	−3.65	176.22	<0.0001
X_2_^2^	1	−3.76	186.87	<0.0001
X_3_^2^	1	−3.56	167.47	<0.0001
X_1_X_2_	1	-	-	-
X_1_X_3_	1	2.11	35.66	<0.0001
X_2_X_3_	1	0.76	4.58	0.0395
Residual	10		8.16	
Lack of Fit	6		6.03	0.2810
Pure Error	4		2.13	
Total	19		3,036.76	
R^2^		0.997		
Adjusted-R^2^		0.995		
E (%)		0.550		

## References

[1] Morton J.F. (1971). The Wax Gourd, Year-Round Florida Vegetable with Unusual Keeping Quality.

[2] Mohd Zaini N.A., Anwar F., Abdul Hamid A., Saari N. (2011). Kundur [*Benincasa hispida* (Thunb.) Cogn.]: A potential source for valuable nutrients and functional foods. Food Res. Int..

[3] Raveendra R.K., Martin P. (2006). Ethnomedicinal Plants.

[4] Yagnik B., Jitendra V., Nurudin J., Nilesh K., Rameshvar P., Natavarlal P. (2009). Antioxidant activity of *Benincasa hispida* on renal ischemia/reperfusion injury. Pharmacology online.

[5] Stevigny C., Rolle L., Valentini N., Zeppa G. (2007). Optimization of extraction of phenolic content from hazelnut shell using response surface methodology. J. Sci. Food Agric..

[6] Bimakr M., Russly A.R., Farah S.T., Noranizan M.A., Zaidul I.S., Ganjloo A. (2012). Antioxidant activity of winter melon (*Benincasa Hispida*) seeds using conventional soxhlet extraction technique. Food Res. Int..

[7] Ghafoor K., Park J., Choi Y.H. (2010). Optimization of supercritical fluid extraction of bioactive compounds from grape (*Vitis labrusca* B.) peel by using response surface methodology. Innov. Food Sci. Emerg. Technol..

[8] Chemat F., Tomao V., Virot M., Semih O. (2008). Ultrasound-assisted Extraction in Food Analysis. Handbook of Food Analysis Instruments.

[9] Cuoco G., Mathe C., Archier P., Chemat F., Vieillescazes C. (2009). A multivariate study of the performance of an ultrasound-assisted madder dyes extraction and characterization by liquid chromatography-photodiode array detection. Ultrason. Sonochem..

[10] Chemat F., Grondin I., Costes P., Moutoussamy L., Sing A.S.C., Smadja J. (2004). High power ultrasound effects on lipid oxidation of refined sunflower oil. Ultrason. Sonochem..

[11] Wang L., Weller C.L. (2006). Recent advances in extraction of nutraceuticals from plants. Trends Food Sci. Technol..

[12] Zenker M., Zenker V.H., Knorr D. (2003). Application of ultrasound-assisted thermal processing for preservation and quality retention of liquid foods. J. Food Prot..

[13] Vinatoru M., Toma M., Mason T.J. (1999). Advances in Sonochemistry.

[14] Romdhane M., Gourdan C. (2002). Investigation in solid-liquid extraction: Influence of ultrasound. Chem. Eng. J..

[15] Vinatoru M. (2001). An overview of ultrasonically assisted extraction of bioactive principles from herbs. Ultrason. Sonochem..

[16] Chemat F., Huma A., Kamran Khan M. (2011). Application of ultrasound in food technology: Processing, Preservation and extraction. Ultrason. Sonochem..

[17] Domingos K.A., Saad E.B., Wilhelm H.M., Ramos L.P. (2008). Optimization of the ethanolysis of *Raphanus sativus* (L. Var.) crude oil applying the response surface methodology. Bioresour. Technol..

[18] Montgomery D.C. (2000). Design and Analysis of Experiments.

[19] Zhang H.F., Yang X.H., Zhao L.D., Wang Y. (2009). Ultrasonic-assisted extraction of epimedin C from fresh leaves of *Epimedium* and extraction mechanism. Innov. Food Sci. Emerg. Technol..

[20] Zou T.B., Wang M., Gan R.Y., Ling W.H. (2011). Optimization of ultrasound-assisted extraction of anthocyanins from mulberry, Using response surface methodology. Int. J. Mol. Sci..

[21] Veličković D.T., Milenović D.M., Ristić M.S., Veljković V.B. (2008). Ultrasonic extraction of waste solid residues from the Salvia sp. essential oil hydrodistillation. Biochem. Eng. J..

[22] Patist A., Bates D. (2008). Ultrasonic innovations in the food industry: From the laboratory to commercial production. Innov. Food Sci. Emerg..

[23] Hossain M.B., Brunton N.P., Patras A., Tiwari B., Donnell C.P., Martin-Diana A.B., Barry-Rayan C. (2012). Optimization of ultrasound assisted extraction of antioxidant compounds from marjoram (*Origanum majorana* L.) using response surface methodology. Ultrason. Sonochem..

[24] Zou Y., Xie C., Gongjian F., Gu Z., Han Y. (2010). Optimization of ultrasound-assisted extraction of melanin from *Auricularia auricular* fruit bodies. Innov. Food Sci. Emerg. Technol..

[25] Zhang Q.A., Zhang Z.Q., Yue X.F., Fan X.H., Li T., Chen S.F. (2009). Response surface optimization of ultrasound-assisted oil extractionfrom autoclaved almond powder. Food Chem..

[26] Zhang Z.S., Wang L.J., Li D., Jiao S.S., Chen X.D., Mao Z.H. (2008). Ultrasound-assisted extraction of oil from flaxseed. Sep. Purif. Technol..

[27] Jadhav D., Rekha B.N., Gogate P.R., Ratho V.K. (2009). Extraction of vanillin from vanilla pods: A comparison study of conventional soxhlet and ultrasound assisted extraction. J. Food Eng..

[28] Hu Q., Pan B., Xu J., Sheng J., Shi Y. (2007). Effects of supercritical carbon dioxide extraction conditions on yields and antioxidant activity of *Chlorella pyrenoidosa* extracts. J. Food Eng..

[29] Rajaei A., Barzegar M., Yamini Y. (2005). Supercritical fluid extraction of tea seed oil and its comparison with solvent extraction. Eur. Food Res. Technol..

[30] Cárcel J.A., Benedito J., Rosselló C., Mulet A. (2007). Influence of ultrasound intensity on mass transfer in apple immersed in a sucrose solution. J. Food Eng..

[31] Zengin G., Cakmak Y.S., Guler G.O., Aktumsek A. (2010). *In vitro* antioxidant capacities and fatty acid compositions of three *Centaurea* species collected from Central Anatolia region of Turkey. Food Chem. Toxicol..

[32] Cai Y.Z., Luo Q., Sun M., Corke H. (2004). Antioxidant activity and phenolic compounds of 112 traditional Chinese medicinal plants associated with anticancer. Life Sci..

[33] Singleton V.L., Orthofer R., Lamuela-Raventos R.M. (1999). Analysis of total phenols and other oxidation substrates and antioxidants by means of Folin-Ciocalteu reagent. Meth. Enzymol..

[34] Chua S.C., Tan C.P., Mirhosseini H., Lai O.M., Long K., Baharin B.S. (2009). Optimization of ultrasound extraction condition of phospholipids from palm-pressed fiber. J. Food Eng..

[35] Weng W.L., Liu Y.C., Lin C.W. (2001). Studies on the optimum models of the dairy product Kou Woan Lao using response surface methodology. Asian Aust. J. Anim. Sci..

[36] Deng Y., Zhao Y.J. (2008). Effects of pulsed-vacuum and ultrasound on the osmodehydration kinetics and microstructure of apples (Fuji). J. Food Eng..

